# DNA binding and cleavage mechanism of DNA topoisomerase VI, an evolutionary counterpart of Spo11/Wadjet/Gabija systems

**DOI:** 10.1063/4.0000853

**Published:** 2025-10-27

**Authors:** Daniel E Richman, Timothy J Wendorff, Fahad Rashid, Curtis Beck, Matthew L Baker, Jonathan M Fogg, Lynn Zechiedrich, James M Berger

**Affiliations:** 1 Dept of Biophysics and Biophysical Chemistry, Johns Hopkins University School of Medicine, Baltimore, MD, USA; 2 Dept of Molecular Virology and Microbiology, Baylor College of Medicine, Houston, TX, USA

## Abstract

Type II topoisomerases resolve DNA supercoiling and chromosome entanglements. Type IIB topoisomerases, exemplified by Top6, are used by plants and archaea to support endoreduplication and cell proliferation, respectively; homologs of Top6 subunits initiate meiotic recombination in eukaryotes and constitute the nuclease portion of Wadjet/Gabija bacterial plasmid defense systems. To understand how such factors act upon native substrates, we determined cryoEM structures of M. mazei Top6 bound to supercoiled DNA in cleaved and uncleaved states. The structures show that Top6 deforms DNA into an ∼80bp loop, explaining its preferential association with supercoiled substrates. Five holoenzyme conformations reveal new interactions and motifs that recognize bent DNA and promote cleavage, as well as an unanticipated tension sensor that couples ATPase disposition to cleavage state activation. Our observations offer unprecedented insight into interdependent structural changes that regulate DNA recognition and cleavage in type II topoisomerases and related systems.

